# The protein-protein interaction ontology: for better representing and capturing the biological context of protein interaction

**DOI:** 10.1186/s12864-021-07827-4

**Published:** 2021-11-16

**Authors:** Mansheng Li, Qiang He, Chunyuan Yang, Jie Ma, Fuchu He, Tao Chen, Yunping Zhu

**Affiliations:** 1 State Key Laboratory of Proteomics, Beijing Proteome Research Center, National Center for Protein Sciences (Beijing), Beijing Institute of Life Omics, 102206 Beijing, China; 2 School of Software and Electrical Engineering, Swinburne University of Technology, 3122 Melbourne, Victoria Australia

**Keywords:** Protein-protein Interaction, PPI Ontology, PPI Annotation, PPI Annotation Extraction

## Abstract

**Background:**

With the rapid increase in the amount of Protein-Protein Interaction (PPI) data, the establishment of an event-centered PPI ontology that contains temporal and spatial vocabularies is urgently needed to clarify PPI biological annotations. In this paper, we propose a precisely designed schema - PPIO (PPI Ontology) for representing the biological context of PPIs.

**Results:**

Inspired by the event model and the distinct characteristics of PPI events, PPIO consists of six core aspects of the information required for reporting a PPI event, including the interactor (who), the biological process (when), the subcellular location (where), the interaction type (how), the biological function (what) and the detection method (which). PPIO is implemented through the integration of appropriate terms from the corresponding vocabularies/ontologies, e.g., Gene Ontology, Protein Ontology, PSI-MI/MOD, etc. To assess PPIO, an approach based on PPIO in developed to extract PPI biological annotations from an open standard corpus “BioCreAtIvE-PPI”. The experiment results demonstrate PPIO’s high performance, a precision of 0.69, a recall of 0.72 and an F-score of 0.70.

**Conclusions:**

PPIO is a well-constructed essential ontology in the interpretation of PPI biological context. The results of the experiments conducted on the BioCreAtIvE corpus demonstrate that PPIO is able to facilitate PPI annotation extraction from biomedical literature effectively and enrich essential annotation for PPIs.

**Supplementary Information:**

The online version contains supplementary material available at 10.1186/s12864-021-07827-4.

## Background

Protein-protein interaction (PPI) plays an important role in biological systems. A series of coordinated actions of groups of protein interactions in molecular assemblies or pathways result in various cellular functions [1]. A proper understanding of PPI can help unveil PPI mechanisms and gain insight into the nature of cellular activities. Despite a wealth of available PPI databases, the temporal or spatial PPI annotations are not fully exploited to comprehensively understand PPI events. The rapid increase in the amount of available PPI data urgently calls for an event-centered PPI ontology (PPIO) that includes the vocabularies relevant to cellular time and space for describing the essential temporal-spatial annotations of PPIs.

Researchers have attempted to interpret PPIs in different ways. Duan et al. [2] tried to describe PPIs in terms of protein states and state transitions. They collected a group of terms to describe protein states and their transitions, including the types of posttranslational modification and the types of ligand bound to the protein during PPI process. Ratsch et al. [3] proposed a method that represents a PPI as an event with a pre- and a post-condition. Hermjakob and Orchard focused on the minimum amount of information required for reporting a molecular interaction experiment and proposed the Proteomics Standards Initiative-Molecular Interaction (PSI-MI) format [4–6] and the Minimum Information about a Molecular Interaction eXperiment (MIMIx) guideline [7]. In addition, many ontologies in the biomedical field have emerged for knowledge representation, data exchange, database design, information retrieval, information extraction, etc. Their subjects range from gene annotation to intricate biological network modeling. Examples include the Gene Ontology (GO) [8, 9] the PSI-MI controlled vocabularies [4, 6] and the Biological Pathway Data Exchange (BioPAX) ontology [10]. However, none of the existing biomedical ontologies and efforts are capable of capturing the temporal and spatial information necessary for understanding the essence of PPI as a molecular event. GO includes abundant vocabularies that describe temporal-spatial characteristics of gene and gene products. However, it does not represent or capture the temporal-spatial attributes specialized in PPI events. PSI-MI was set up as a standard data model for the representation, exchange, and integration of PPI experimental data [4]. It mainly included the terms of experimental information, chemical information and pharmaceutical information of PPIs. It has been widely adopted by many research institutes, including the IMEx consortium [11]. A PPI is inherently a biomolecular event with temporal-spatial attributes. An ontology is in urgent need that incorporates vocabularies for the temporality and spatiality of PPI events. To capture the essence of PPI, this paper proposes an event-centered ontology, namely PPIO, to comprehensively represent the context of PPI, especially the temporal and spatial perspectives.

Another key promises of this ontology is its potential ability to facilitate automatic information extraction. This is of particular significance to the annotation of PPI scattered across rapidly-increasing biomedical literature. Hence, we evaluated the efficiency of PPIO to extraction the PPI annotations on an open standard corpus “BioCreAtIvE-PPI”.

This paper proposes PPIO, an ontology constructed based on the event model for describing PPIs. In addition, the PPIO-based process for extracting PPI annotations from literature is described and discussed.

## Methods

### Design and construction

In general, PPIO was designed to contain the temporal-spatial information on PPI events. In addition, the selection of ontology terms must fulfill the needs for further information extraction and text mining that aim to identify PPI annotations in biomedical literature. Thus, there were two principles for the construction of PPIO. First, it was required to manifest the biological knowledge of PPIs. Second, it was required to be suitable for PPI annotation extraction. Accordingly, PPIO was constructed in three mainly steps: (1) setting the information scope of the PPIO based on event model; (2) reusing existing biological ontologies and nomenclatures for PPIO construction; and (3) assessing PPIO through extracting PPI annotations from literature.

**Fig. 1:**
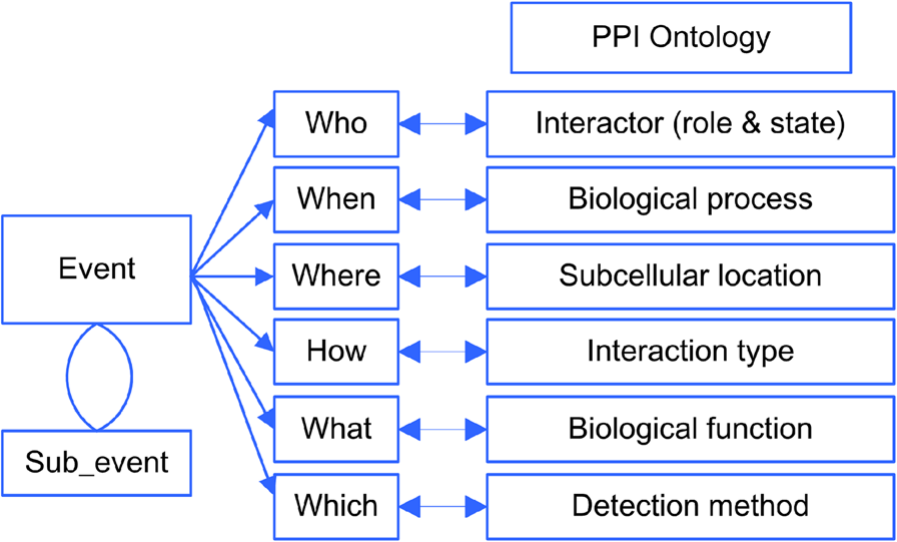
The event model and the structure of PPIO

Event Model. PPI is essentially a molecular event that occurs under particular conditions. The annotations of a PPI are similar to the elements that define an event. The event model [12], which deals with the notion of reified events, was employed to outline the components of PPIO. According to the referred event model, there are five key elements that the majority of events have in common, including (1) active agents; (2) a time point; (3) a location; (4) factors; and (5) products. Based on this event model, a PPI can be described as a biological event with temporal-spatial attributes represented by the interactor (who), the biological process (when), the subcellular location (where), the interaction type (how), the biological function (what) and the detection method (which, the witness/evidence to support the PPI event.), as illustrated in Fig. 1. These attributes are considered the minimum annotation categories required for the description of a PPI.

Integrating related ontologies. The elementary content and structure of PPIO was curated manually based on the textbook and the terminologies from two public biomedical ontology resources, i.e., OBO Foundry [13] and NCBO BioPortal [14]. The terms and sub-ontologies that fulfill the construction principles were integrated into PPIO while maintaining the references of the original source. Table 1 lists all the related sources and their corresponding information scopes. Together, the terms and sub-ontologies constitute the six cardinal requirements for the minimal core information of PPI, as illustrated in Fig. 1. The URLs of these ontologies are provided in Table S1 [See Additional file 1]. The critical ontologies referred to by PPIO are summarized as follows.

**Table 1:** Existing ontological resources related to PPIO

Name	Domain
Gene Ontology (GO) [8]	Biological process, Cellular component and Molecular function.
Protein Ontology (PRO) [15]	Protein family, codeing gene, sequence and modification.
Genetic Regulation Ontology (GRO) [16]	Gene regulatory processes modelling.
Proteomics Standards Initiative – Molecular Interactions (PSI-MI) [4, 6]	Molecular interaction experiment annotation.
Proteomics Standards Initiative – Protein Modification (PSI-MOD) [17]	Controlled vocabularies for representation of protein modification.
INOH Ontology [18]	Biological event and biological pathway data annotation.
WordNet [19]	On-line lexical reference system for English.
BioPAX Ontology [20]	Biological pathway data exchange.
Systems Biology Ontology (SBO) [21]	Biological system modelling.

GO [8, 9] is one of the most popular ontologies in the biological domain. It consists of the biological process sub-ontology (BP), the cellular component sub-ontology (CC), and the molecular function sub-ontology (MF). GO has been widely used to annotate the functions of genes and gene products. Although it was not designed for annotating molecular interactions, it is of great value as a reference for PPIO due to its rich vocabularies for describing biological processes.

PSI-MI [4, 6], developed by the Proteomics Standards Initiative (PSI), is a widely adopted standard for PPI data annotation, mainly composed of experimental information. The latest version of PSI-MI controlled vocabularies (CV) is 3.0, which contains 21 sub-ontologies, 1,572 terms and multiple types of relationships between terms, e.g., “is_a”, “contains”, “part_of”, “derives_from” and “has_functional_parent”, etc.

INOH Ontology [18] is a pathway annotation ontology consisting of structured and controlled vocabularies of pathway-centric biological events. According to INOH Ontology, biological events can be divided into five hierarchical levels: physiological events, organism events, cellular events, molecular events and environmental events. At the molecular event level, molecular interactions are further divided into binding, co-localization, genetic interaction, dissociation, and so on.

BioPAX [10] is a pathway exchange language for biological pathway data. It describes the biological network at three levels, i.e., physical entity level, interaction level and pathway level.

**Fig. 2:**
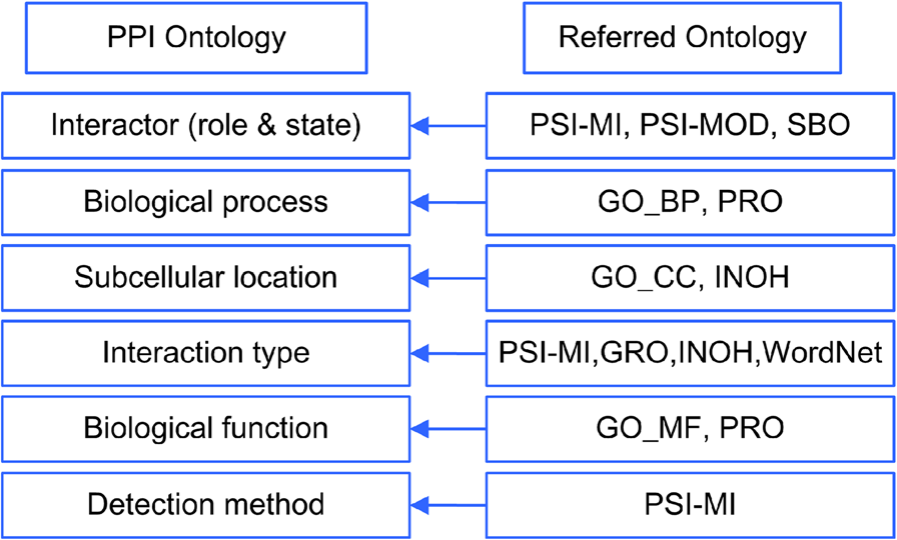
The construction strategies of the PPIO. GO_BP, the Biological Process sub-ontology of GO; GO_MF, the Molecular Function sub-ontology of GO; GO_CC, the Cellular Component sub-ontology of GO

We used OBO-Edit [22] to compile PPIO. The strategy used to construct PPIO is illustrated in Fig. 2. The terms in each class were derived from the corresponding ontologies. Besides whether the terms manifest the knowledge of PPI, the appearances of terms in literature were also taken into account to facilitate PPI annotation extraction. PubMed literature were retrieved used candidate terms. Corresponding statistics were obtained through the PubMed’s online API namely PubMed eUtils [23]. Terms that had zero associated articles under the exact match retrieval strategy were removed. Finally, a reference ID was kept for each selected terms to trace its source. The hierarchical relationships and their definitions were defined or inherited from the referred ontologies. Specifically, the terms in interactor sub-ontology were retrieved from PSI-MI, PSI-MOD [17] and SBO [21]. The terms in the biological process sub-ontology were derived from the biological process sub-ontology of GO. The terms in the cellular component sub-ontology of GO and INOH were selected and utilized in the construction of PPIO subcellular location sub-ontology. The complex-related terms in the cellular component sub-ontology of GO were filtered out because they are not actual sub-cellular locations. The corresponding terms in the molecular function sub-ontology of GO and Protein Ontology (PRO) [15] were adopted to construct biological sub-ontology of PPIO. Experiment-related terms in PSI-MI were reused to construct the detection method sub-ontology. The terms that represent the relationships between entities in PPIO were derived from the OBO Relation Ontology (RO) [24]. The interaction type sub-ontology of PPIO was built using a top-down and bottom-up combined approach illustrated in the next paragraph.

Building the interaction type sub-ontology of PPIO. Existing ontologies rarely model verbs (including nominal verbs) as relations between proteins. However, particular verbs play an important role as a central connecting element between proteins. Hence, the verbs that indicate particular types of interactions were collected from related works (see Table S2 in Additional file 2) and organized into hierarchical structure. The following two steps were took to organize this sub-ontology. First, we employed a top-down approach to classify the interaction type at the most general level. To properly classify different interaction types, four molecular interaction type-related ontologies were summarized, including PSI-MI, INOH Ontology, GRO and BioPAX. The top interaction types from the PSI-MI included genetic interaction, co-localization, and association (physical association), whereas the INOH ontology defined four interaction types, i.e., binding, co-localization, genetic interaction, and dissociation. GRO provides a “physical interaction” branch within its “occurrent” class. BioPAX included six terms within its interaction sub-class, including control, conversion, molecular-association, co-occurrence, equivalent class and genetic. The classifications of these ontologies were summarized to constitute the sublevel of interaction type sub-ontology in PPIO. Secondly, a bottom-up approach was employed to categorize the words denoting interactions into the top-level types of interactions. To categorize the collected words into the proper interaction types and to confirm their hierarchy, the words were pre-clustered based on their semantic similarity. The Java WordNet Similarity Library (JWSL) [25] was employed to calculate the similarity between the interaction words based on their existing semantic relationships from WordNet [19].

### Evaluating PPIO

To assess PPIO for its structure and functional features, we first applied it to capture PPI annotations from literature, which was conducted on an open standard corpus, annotating extracted PPIs based on PPIO and assessing the performance. Then, we employed PPIO to navigate PPI information.

Annotating PPIs based on PPIO. To annotate extracted PPIs, a PPIO-based approach was proposed to identify and assign PPIO terms that exist in the same sentence with the target PPI. The co-occurrence of PPI and PPIO term in one sentence suggests that the term represents a type of annotations of the PPI.

*Corpus and preprocessing.* A corpus named “BioCreAtIvE-PPI” [26] (See Table S3 in Additional file 3) was used to evaluate the efficacy of PPIO-based annotation extraction. This dataset originated from the BioCreAtIvE Task [27] corpus. A total of 173 sentences, which contained 255 interactions, were randomly selected from the BioCreAtIvE corpus by the original PPI curator. Based on these sentences which contained at least one PPI, six aspect additional annotations of PPI were curated manually by individual annotators according to the PPIO schema. In total, 71 Roles/Status of interactors, 91 biological processes (BPs), 17 subcellular locations (SCLs), 274 interaction types (ITs), 53 biological functions (BFs) and 43 detection methods (DMs) of PPIs were labeled on the original “BioCreAtIvE-PPI” corpus. This innovate curated corpus (See Table S4 in Additional file 4) was then used in the evaluation procedure. In order to create the reference corpus, the annotators were asked to keep in mind the breadth and depth of PPIO and to consider not only the superclass concepts but also their corresponding sub-class concepts as well as their synonyms for annotation.

*Assigning annotations to related PPIs based on PPIO.* We used the terms of PPIO as a dictionary for PPI annotation extraction. A PPIO-based approach which consists of three steps was proposed to accomplish the annotation task. First, a string matching algorithm was applied to recognize all the case-insensitive names and synonyms of the PPIO terms in sentences containing PPIs. Then, in the case of multiple matches, the longest match was selected. For instance, when the terms “regulation” and “regulation of transcription” were both identified, “regulation of transcription” was selected. Finally, the results were validated manually and the performance of the PPIO-based approach was evaluated using the curated corpus described above. The evaluation process focused on the performance comparison between the automatically assigned corpus and the manually curated corpus. Three commonly used features, i.e., precision, recall and F-score, were used to measure the performance of the PPI annotation extraction:

$${\text{Precision = }}\frac{{{\text{True Positive}}}}{{{\text{True Positive}}+{\text{ False Positive}}}}{\text{ }}……{\text{ }}……{\text{ }}……{\text{ (1)}}$$

$${\text{ Recall = }}\frac{{{\text{ True Positive }}}}{{{\text{True Positive + False Negetive }}}}{\text{ }}…{\text{ }}……{\text{ }}……{\text{ (2)}}$$

$${\text{ F-score = }}\frac{{{\text{ 2 }} \times {\text{ Precision }} \times {\text{ Recall }}}}{{{\text{Precision }}+{\text{ Recall }}}}{\text{ }}……{\text{ }}……{\text{(3)}}$$

where true positive is the number of entities that were found by the PPIO-based text mining system, and those matched the annotations in the curated corpus, false positive is the number of entities that were automatically assigned by the PPIO-based text mining system but could not be matched to any annotations in the manually curated corpus, and false negative is the number of entities that were not found by the PPIO-based approach when compared with the manually curated annotations. Higher precision, recall and F-score indicate high performance. Further details of evaluation material and methods are provided in Additional file 13.

## Results

### Structure and statistics of PPIO

Component of the PPIO. To better represent the temporal and spatial PPI information, we proposed an event-centered PPI ontology (PPIO) including six sub-ontologies, i.e., interactors, biological processes, subcellular locations, interaction types, biological functions and detection methods.

The interactor sub-ontology mainly consists of the terms of the participant’s special properties that appear only in the interaction process. This class is divided into the “biological role” and “protein state” subclasses. The “biological role” subclass describes the role played by the protein, e.g., “regulator” or “acceptor”, whereas the “protein state” subclass describes the state of the protein, e.g., modification state, phosphorylation or ubiquitination, when PPI occurs.

The biological process sub-ontology of PPIO is used to illustrate the biological process that PPI participates in. The connotation of biological process of PPIO comes from the definition of biological process in GO, i.e., “A biological process is accomplished by a particular set of molecular processes carried out by specific gene products, often in a highly regulated manner and in a particular temporal sequence”[28, 29]. In general, a biological process consists of a series of events accomplished by one or more ordered assemblies of molecular activities [28]. The terms in this sub-ontology representing the temporal attributes of PPI are mainly derived from the biological process sub-ontology of GO.

The subcellular location sub-ontology includes the terms of locations where PPIs occur. Studies [30–32] have shown that most proteins have multiple locations. They interact with each other in different locations, performing different functions. The first level on this sub-ontology includes three parts, i.e., “extracellular region”, “intracellular region” and “membrane”.

The interaction type sub-ontology represents the mechanisms of PPI. It has been shown that various types of interactions exist among proteins [33]. A protein may interact for a long time to form part of a protein complex, or carry another protein for a while, e.g., from cytoplasm to nucleus or vice versa in the case of the nuclear pore importins. It may also interact transiently with another protein merely to modify it, e.g., a protein kinase adds a phosphate to a target protein [34]. This sub-ontology of PPIO predominantly consists of words that indicate particular types of interactions.

The biological function sub-ontology represents the effects produced by PPIs. The terms in the molecular function sub-ontology of PPIO are inherited from corresponding sub-ontology of GO and PRO.

The detection method sub-ontology refers to the experimental strategies used to detect PPI, such as yeast two hybrid, co-immunoprecipitation and tandem affinity purification.

The relationships between entities in PPIO include “is_a”, “part_of” and “proper_part_of”. Relationship “is_a” is the most fundamental relationship in the PPIO and is used to indicate the relationship between a specific class and a more general one. Relationship “part_of” is used to indicate the relationship between the temporal or spatial part and the whole object, while the “proper_part_of” relationship is used to relate properties to the object.

**Fig. 3:**
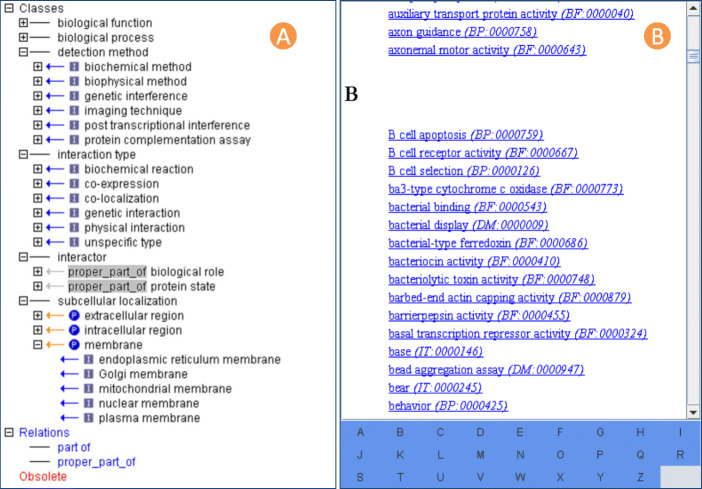
The hierarchical structure and glossary of PPIO. **(A)** The hierarchical structure of PPIO. **(B)** The glossary of PPIO terms. The open-source ontology editor OBO-Edit (version 2.3.1) was used to display the PPIO hierarchical tree and term glossary

Structure of the PPIO. The hierarchical structure and glossary of PPIO are shown in Fig. 3 (A) and Fig. 3 (B). The global view of PPIO concepts is displayed in a hierarchical structure in Fig. 3 (A). PPIO in OBO format can be downloaded from http://ppio.hupo.org.cn/download.jsp and browsed in the ontology editor named OBO-Edit (version 2.3.1).

Statistics of terms in sub-ontologies of PPIO. The statistics of sub-ontology terms are shown in Table 2. The numbers of terms in the interactor, the biological process, the subcellular location, the interaction type, the biological function and the detection method sub-ontology are 82, 1,033, 66, 257, 923 and 216, respectively. Moreover, the statistics of each sub-ontology terms derived from external sources are given: (1) For the interactor sub-ontology, 29 terms from PSI-MOD describing protein posttranslational modifications and 30 terms from PSI-MI were selected to constitute the major part of the “protein state” branch; (2) 17 terms from both PSI-MI and SBO were selected to construct the “biological role” branch; (3) 1,022 terms from the biological process sub-ontology of GO and 11 terms from PRO were selected to construct the biological process sub-ontology of the PPIO; (4) The subcellular location sub-ontology consists of three branches, namely “extracellular region”, “intercellular region” and “membrane”. A total of 54 terms in this sub-ontology were selected from the cellular component sub-ontology of GO and another 12 terms were derived from the location sub-ontology of INOH; (5) 905 terms from the molecular function sub-ontology of GO and 18 terms from PRO were selected to construct the biological function sub-ontology of the PPIO; (6) The detection method sub-ontology contains 216 terms derived from PSI-MI.

**Table 2:** Statistics of sub-ontologies in PPIO

Component	Sub-ontology	Number of terms
Who	Interactor (Role and State)	82
When	Biological Process	1,033
Where	Subcellular Location	66
How	Interaction Type	257
What	Biological Function	923
Which	Detection Method	216
All entities		2,577

**Fig. 4:**
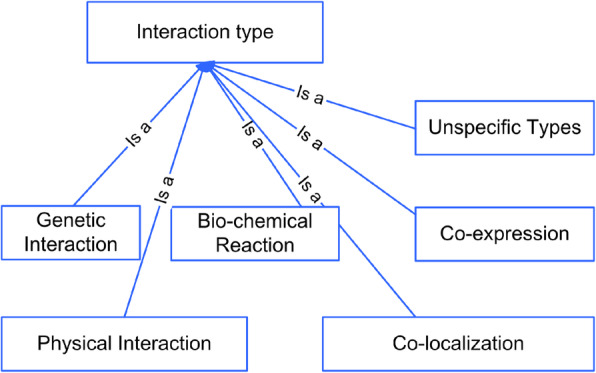
The hierarchical structure of top-level categories in interaction type sub-ontology of PPIO

Interaction type sub-ontology. As discussed in the “*Building the interaction type sub-ontology of PPIO*” section, the top-level categories of the interaction type sub-ontology can be classified into six interaction sub-categories according to existing ontologies, i.e., genetic interaction, physical interaction, bio-chemical reaction, co-expression, co-localization and unspecific types (Fig. 4). The words denoting interactions were categorized into the bottom level of interaction type sub-ontology. Finally, 247 verbs and 92 nouns denoting PPIs were confirmed after removing the redundant words. These words are provided in Table S5 [See Additional file 5].

### Evaluation of PPIO-based PPI annotation extraction

Ontologies can be used in a variety of ways, e.g., knowledge representation, data exchange, database design, and information retrieval/extraction. Most particular ontologies are designed for a specific application area. PPIO is specifically constructed for PPI annotation extraction,

We evaluated the efficacy of the PPI annotation extraction on the dataset manually curated from the “BioCreAtIvE-PPI” corpus. The results are shown in Table 3, the PPIO-based method achieved precisions of 0.55, 0.67, 0.64, 0.75, 0.64 and 0.88, recalls of 0.76, 0.79, 0.82, 0.65, 0.87, 0.67, and F-scores of 0.64, 0.72, 0.72, 0.70, 0.74, and 0.76 in the interactor, the biological process, the subcellular location, the interaction type, the biological function, and the detection method annotation category, respectively. Overall, its precision, recall and F-score are 0.69, 0.72 and 0.70 (see Table S6-12 in Additional file 6,7,8,9,10,11,12 for more detail of evaluation results).

**Table 3:** The performance of the PPIO-based approach on test dataset

Annotation categories	Precision	Recall	F-score
Interactor (Role and State)	0.55	0.76	0.64
Biological Process	0.67	0.79	0.72
Subcellular Location	0.64	0.82	0.72
Interaction Type	0.75	0.65	0.70
Biological Function	0.64	0.87	0.74
Detection Method	0.88	0.67	0.76
Total	0.69	0.72	0.70

### Application of PPIO to navigate PPI data

**Fig. 5:**
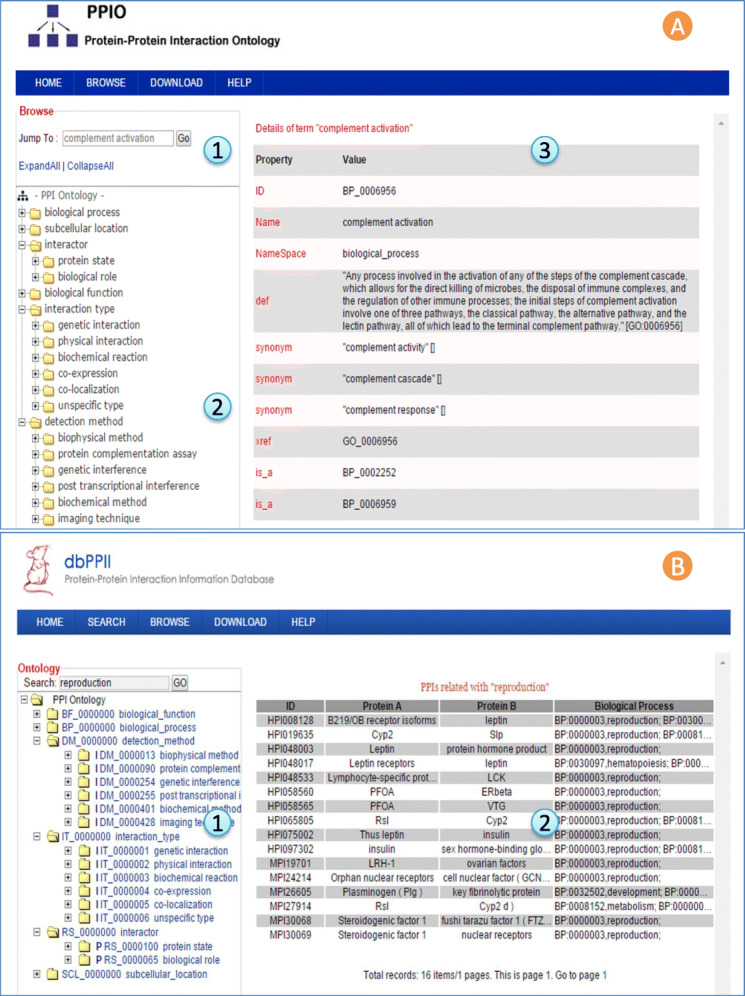
Interface of PPIO Browser and “mouse” related PPI information database. (**A**) PPIO Browser. PPIO hierarchy and term details are displayed using a web server. The browser is customized to search ontology by term name (A1) and display the ontology hierarchy (A2) and term details (A3). (**B**) “Mouse” related PPI information database (dbPPII). A global view for navigating the whole database based on PPIO tree (B1). PPIs related with “reproduction” functional annotation (B2)

We built an ontology browser for user to explore each sub-ontology of PPIO, which is accessible at http://ppio.hupo.org.cn/index.jsp. A screenshot of PPIO in a browser is given in Fig. 5 (A). Also, a PPI database named dbPPII (http://ppii.hupo.org.cn) was designed and implemented to store and display rich PPI annotations of mouse we have mined. A snapshot of dbPPII’s user interface is shown in Fig. 5 (B). The PPIO tree is used as a global view for navigating the whole database. PPI annotation information can be displayed and searched vertically based on the hierarchical PPIO. PPIs with the same annotation can be collected with keyword query or PPIO term linkage. In this way, precise function-related PPIs can be unveiled which will help with the construction and analysis of PPI networks.

## Discussion

PPIO was inspired by the event model and was designed to represent PPI context and facilitate PPI annotation extraction. The specific requirements for PPIO differentiate it from other OBO ontologies. Compared with GO, which consists of the biological process sub-ontology, the cellular component sub-ontology, and the molecular function sub-ontology, PPIO is not used to annotate the node (gene and its product) as GOA mission [35], but to annotate the edges (interactions) instead. It is more specific and accurate to functionally annotate PPIs than to annotate proteins. The reason is that proteins always perform specific functions through interacting with other proteins in certain processes and subcellular locations. The selection of appropriate terms for representing the context of PPI makes PPIO more suitable for PPI annotation extraction than GO. Compare with PSI-MI, which is constructed to annotate experimental PPI data, PPIO, which is designed based on the principles of the event model, with a focus on the essential temporal-spatial information of PPI. While PSI-MI is concerned with up to 21 types of information surrounding PPI experimental data, PPIO focuses on the core biological knowledge of PPI which includes six space-time elements (who, when, where, what, how, which) of biomolecular events. Thus, while PSI-MI is widely acknowledged as a community standard for annotating experimental context of PPI data, PPIO can be used as an extension to annotate the biological context of PPIs. It is a streamline and essential ontology in the interpretation of PPI context.

PPIO can be utilized in many areas, such as PPI data annotation and integration (as an extension of community standard), PPI networks analysis and PPI annotation extraction. The interaction verbs and nouns included in the interaction type sub-ontology have contributed to improving the performance of PPI extraction approach [36, 37]. It can also be used to infer the interaction type of a PPI based on the hieratical ontology. The PPIO-based extraction approach was evaluated on a manually curated corpus. It obtained a precision of 0.69, a recall of 0.72 and a F-score of 0.70 on average across all annotation categories. This indicates the high performance of PPIO. PPIO serves as a controlled vocabulary resource for PPI annotation in biomedical literature, which paves the way for more sophisticated knowledge-intensive text mining tasks. To our best knowledge, this is the first attempt to propose an innovative ontology for representing in-depth biological annotations of PPI and to enrich them using a literature-based method. The PPIs with rich annotations are useful for integrating and constructing PPI networks under various conditions (dynamic PPI networks).

In real-world applications, the extraction of PPI annotations from biological literature goes through at least three phases, i.e., entity recognition (NER) for protein name identification, relation extraction (RE) for PPI detection, and term recognition (TR) for PPI annotation assignment. These operations are error prone, which consequently jeopardizes the performance of PPI annotation extraction, especially in the TR phase. Thus, there is still room for improvement.

In the future, PPIO will be expanded with more apt terms and a series of tools will be developed to support the use of PPIO. The PPIO-based approach will be improved by scaling up PPIO terms and developing more efficient algorithms for term recognition.

## Conclusions

This paper presents the construction process of PPIO, a conceptual model for PPI annotation, which involves temporal-spatial information of PPI at the cellular level. PPIO focuses on the roles and states of interactor, the biological process, the subcellular location, the type of interaction, the biological function, and the detection method of PPI. The results of the experiments conducted on “BioCreAtIvE-PPI” corpus demonstrate that PPIO is able to facilitate PPI annotation extraction from biomedical literature effectively and enrich essential annotation for PPIs. It also indicates that PPIO is an essential schema in the interpretation of PPI context.

## Supplementary information

Additional file 1 Table S1. Conceptual resources related to PPI Ontology.

Additional file 2 Table S2. Summary of PPI denoting words collected for PPI ontology construction.

Additional file 3 Table S3.The sentences with protein-protein interactions in “BioCreAtIvE PPI” corpus.

Additional file 4 Table S4. The sentences with PPIs and PPI annotations mined manually from “BioCreAtIvE PPI” corpus.

Additional file 5 Table S5. Verbs and nouns used to construct Interaction Type sub-ontology.

Additional file 6 Table S6. The sentences with PPIs and biological process information extracted from “BioCreAtIvE PPI” corpus by the PPIO-based method.

Additional file 7 Table S7. The sentences with PPIs and subcellular location information extracted from “BioCreAtIvE PPI” corpus using the PPIO-based method.

Additional file 8 Table S8. The sentences with PPIs and biological function information extracted from “BioCreAtIvE PPI” corpus by the PPIO-based method.

Additional file 9 Table S9. The sentences with PPIs and interaction type information extracted from “BioCreAtIvE PPI” corpus by the PPIO-based method.

Additional file 10 Table S10. The sentences with PPIs and proteins’ role & state information extracted from “BioCreAtIvE PPI” corpus by the PPIO-based method.

Additional file 11 Table S11. The sentences with PPIs and detection method information extracted from “BioCreAtIvE PPI” corpus by the PPIO-based method.

Additional file 12 Table S12. The performance of dictionary-based method on test dataset.

Additional file 13 Supplementary Material and Methods.

## Data Availability

PPIO browser is accessible at http://ppio.hupo.org.cn/index.jsp.

## Abbreviations

PPI: Protein-Protein Interaction
PPIO: Protein-Protein Interaction Ontology
GO: Gene Ontology
BP: Biological Process
CC: Cellular Component
MF: Molecular Function
SCL: Subcellular Location
BF: Biological Function
IT: Interaction Type
GOA: Gene Ontology Annotation
PRO: Protein Ontology
GRO: Genetic Regulation Ontology
PSI-MI: Proteomics Standards Initiative - Molecular Interactions
PSI-MOD: Proteomics Standards Initiative - Protein Modifications
SBO: Systems Biology Ontology
CV: Controlled Vocabularies
NER: Named Entity Recognition
RE: Relation Extraction
TR: Term Recognition
