# Supplementary material for: The protein-protein interaction ontology: for better representing and capturing the biological context of protein interaction
Source: BMC Genomics. 2021 Nov 16;22(Suppl 5):544. doi: 10.1186/s12864-021-07827-4 (PMC8596923; doi:10.1186/s12864-021-07827-4)
Supplement: Supplementary file 1 — Table S1. Conceptual resources related to PPI Ontology. [file 12864_2021_7827_MOESM1_ESM.docx]

**Table S1.** Conceptual resources related to PPI Ontology

| Name | URLs |
| --- | --- |
| Gene Ontology (GO) | http://www.geneontology.org/GO.downloads.ontology.shtml |
| Protein Ontology (PRO) | http://pir.georgetown.edu/pro/pro.shtml |
| Genetic Regulation Ontology (GRO) | http://www.ebi.ac.uk/Rebholz-srv/GRO/GRO.html |
| The HUPO PSI's molecular interaction format (PSI-MI) | http://psidev.cvs.sourceforge.net/viewvc/psidev/psi/mi/rel25/data/psi-mi25.obo |
| Proteomics Standards Initiative – Protein Modification(PSI-MOD) | Community standard for representation of protein modification data. |
| INOH Ontology | http://www.inoh.org/ontologies/EventOntology.obo |
| WordNet | http://wordnet.princeton.edu/ |
| BioPAX ontology | http://www.biopax.org/release/biopax-level3.owl |
| Systems Biology Ontology (SBO) | http://www.ebi.ac.uk/sbo/ |
