# Supplementary material for: The protein-protein interaction ontology: for better representing and capturing the biological context of protein interaction
Source: BMC Genomics. 2021 Nov 16;22(Suppl 5):544. doi: 10.1186/s12864-021-07827-4 (PMC8596923; doi:10.1186/s12864-021-07827-4)
Supplement: Supplementary file 2 — Table S2. Summary of PPI denoting words collected for PPI ontology construction. [file 12864_2021_7827_MOESM2_ESM.docx]

**Table S2.** Summary of PPI denoting words collected for PPI ontology construction

| Source | Number of Words | Source | Number of Words |
| --- | --- | --- | --- |
| BioLexicon | 759 verbs | Temkin *et al.,* 2003 | 53 verbs |
| iHop | 74 verbs | Huang *et al.,* 2004 | 30 verbs |
| RelEx | 175 verbs | Santos *et al.,* 2008 | 25 verbs |
| LLL05 | 96 verbs, 37 nouns | Hakenberg *et al.,* 2006 | 520 verbs, 160 nouns |
