# Supplementary material for: The protein-protein interaction ontology: for better representing and capturing the biological context of protein interaction
Source: BMC Genomics. 2021 Nov 16;22(Suppl 5):544. doi: 10.1186/s12864-021-07827-4 (PMC8596923; doi:10.1186/s12864-021-07827-4)
Supplement: Supplementary file 5 — Table S5. Verbs and nouns used to construct Interaction Type sub-ontology. [file 12864_2021_7827_MOESM5_ESM.docx]

**Table S5.** Verbs and nouns used to construct Interaction Type sub-ontology

| Type (level 1) | Subtype (level 2) | Specification(level 2/level 3) |
| --- | --- | --- |
| Genetic Interaction | Positive regulation | Verbs and nouns denoting interaction   \| abolish  abrogate  absorb  accelerate  accept  accumulate  acetylate  acquire  act  activate  active  adapt  add  adhere  affect  aggregate  alter  alternate  amplify  anchor  antagonize  approve  arrest  assemble  assist  associate  attach  attack  attenuate  augment  autophosphorylate  autoregulate  base  bear  bind  biotinylate  block  blockable  bond  bound  break  carboxylate  catalyse  catalyze  cause  cleavage  cleave  cluster  co-activate  co-exist  co-express  co-immunoprecipitate  co-immunopurify  co-infect  co-localize  co-operate  co-precipitate \| co-purificate  co-purify  compete  complex  comprise  conjugate  connect  consist  contact  contain  control  converse  convert  cooperate  coprecipitate  copurificate  correlate  counteract  couple  create  cross-link  cross-talk  deacetylate  deactivate  decline  deconjugate  decrease  degradative  degrade  delete  demethylate  depend  dephosphorylate  deplete  deposit  depress  derive  destabilize  destruct  detach  dim  dimerize  dimerize  diminish  direct  disassemble  discharge  disrupt  dissociate  dock  down-regulate  effect  elevate  enable  encode  encompass  enhance \| enrich  evoke  exert  exhibit  expand  expose  express  form  fuse  generate  glucosylate  glycosylate  hasten  heterodime  hydrolyse  hyper-express  hyper-phosphorylate  imitate  immuno-blot  immuno-labele  immuno-precipitate  immuno-precipitate  immuno-react  impair  import  improve  inactivate  incite  include  increase  induce  infect  influence  inhibit  initiate  inject  interact  interfere  interplay  interrupt  join  label  ligand  ligate  link  localise  localize  mediate  methylate  migrate  mobilise  moderate  modify  modulate  mono-ubiquitinate  multi-ubiquitinate  myogenesis \| neutralize  obstruct  operate  oppose  overexpress  overproduce  oxidise  oxidize  pair  participate  phosphorylate  poly-ubiquitinate  potentiate  precipitate  prevent  process  produce  prohibit  promote  protect  pyruvate  react  recept  recognise  recognize  recruit  reduce  regulate  relate  release  remove  repair  replace  repress  require  respond  response  responsive  restrict  result  secrete  sever  signal  splice  stabilize  sustain  stimulate  substitute  subunit  sumoylate  suppress  supress  suspend  synergize  synthesise  synthesize  target \| tether  trans-regulate  transacetylate  transactivate  transcribe  transcript  transduce  transfer  transform  translate  translocate  transport  treat  trigger  ubiquitinate  up-regulate  utilise  utilize  yield  =====  abolishment  abrogation  acceleration  accumulation  acetylation  activation  activity  addition  affection  amplification  apparatus  assembly  association  attachment  augmentation  augmention  binding  bond  complex  complexes  conjugation  control  conversion  cross-linkage  deconjugation  degradation  demethylation  dephosphorylation  depletion  destabilization  destruction  detachment  disruption  down-regulation  effect  elevation \| expansion  exposion  expression  formation  hyper-expression  immuno-reactivity  inactivation  induction  infection  influence  inhibition  interaction  ligand  ligase  ligation  mediation  methylation  modification  modulation  mono-ubiquitination  multi-ubiquitination  myogenesis  obstruction  over-expression  participation  phosphorylation  poly-ubiquitination  precipitation  prevention  production  promotion  proteolysis  reaction  recognition  recruitment  reduction  regulation  replacement  repression  requirement  sequestration  stabilization  stimulation  substitution  sumoylation  suppression  synthese  synthesis  transacetylation  transactivation  transcription  transduction  treatment  ubiquitination  up-regulation \| \| --- \| --- \| --- \| --- \| --- \| --- \| |
|  | Negative regulation |  |
|  | Affect |  |
|  | … |  |
| Physical Interaction | Bind |  |
|  | Crosslink |  |
|  | Attach |  |
|  | Adhere |  |
|  | Add |  |
|  | Complex |  |
|  | … |  |
| Bio-chemical Reaction | Enzymatic reaction |  |
|  | Modification |  |
|  | Polymerization |  |
|  | … |  |
| Co-expression | Assembly |  |
|  | Co-express |  |
|  | Co-exist |  |
|  | … |  |
| Co-localization | Co-localize |  |
|  | Co-precipitate |  |
|  | … |  |
| Unspecific Types | Associate |  |
|  | Interact |  |
|  | Transport |  |
|  | … |  |
