# Supplementary material for: The protein-protein interaction ontology: for better representing and capturing the biological context of protein interaction
Source: BMC Genomics. 2021 Nov 16;22(Suppl 5):544. doi: 10.1186/s12864-021-07827-4 (PMC8596923; doi:10.1186/s12864-021-07827-4)
Supplement: Supplementary file 12 — Table S12. The performance of dictionary-based method on test dataset. [file 12864_2021_7827_MOESM12_ESM.docx]

**Table S12.** The performance of PPIO-based approach on test dataset

| Annotation categories | Number of PPI annotation curated by expert | Number of PPI annotation extracted by system | | | Precision | Recall | F-score |
| --- | --- | --- | --- | --- | --- | --- | --- |
|  |  | TP | FP | TP+FP |  |  |  |
| Interactor Role&State | 71 | 54 | 44 | 98 | 0.55 | 0.76 | 0.64 |
| Biological Process | 95 | 75 | 37 | 112 | 0.67 | 0.79 | 0.72 |
| Subcellular Location | 17 | 14 | 8 | 22 | 0.64 | 0.82 | 0.72 |
| Interaction Type | 252 | 163 | 53 | 216 | 0.75 | 0.65 | 0.70 |
| Biological Function | 53 | 46 | 26 | 72 | 0.64 | 0.87 | 0.74 |
| Detection Method | 33 | 22 | 3 | 25 | 0.88 | 0.67 | 0.76 |
| Total | 521 | 374 | 171 | 545 | 0.69 | 0.72 | 0.70 |

*Note:* TP= True Positive, FP= False Positive.
