# Supplementary material for: The protein-protein interaction ontology: for better representing and capturing the biological context of protein interaction
Source: BMC Genomics. 2021 Nov 16;22(Suppl 5):544. doi: 10.1186/s12864-021-07827-4 (PMC8596923; doi:10.1186/s12864-021-07827-4)
Supplement: Supplementary file 13 — Supplementary Material and Methods. [file 12864_2021_7827_MOESM13_ESM.docx]

# Supplementary Material and Methods

## Datasets

A corpus named “BioCreAtIvE-PPI” (http://www2.informatik.hu-berlin.de/~hakenber/corpora/#bc, was used to evaluate the efficacy of PPIO-based annotation extraction. This dataset originated from the BioCreAtIvE Task corpus (<http://www.biocreative.org/>) [1]. A total of 173 sentences, which contained 255 interactions, were randomly selected from the BioCreAtIvE corpus by the original PPI curator. Based on these sentences which contained at least one PPI, six aspect additional annotations of PPI **were curated manually** by individual annotators according to the PPIO schema. Totally, 71 Roles Or Status of interactors, 91 BPs, 17 SCLs, 274 ITs, 53 BFs and 43 DMs of PPIs were labeled on the original “BioCreAtIvE-PPI” corpus. This novel curated corpus (See Table S4 in Additional file 4) was then used in the evaluation procedure. In order to create the reference corpus, the annotators were asked to keep in mind the breadth and depth of PPIO and to consider not only the superclass concepts but also their corresponding sub-class concepts as well as their synonyms for annotation.

## PPIO-based approach

To annotate extracted PPIs, a PPIO-based approach was proposed to identify and assign PPIO terms that exist in the same sentence with the target PPI. The co-occurrence of PPI and PPIO term in one sentence suggests that the term represents a type of annotations of the PPI. We used the terms of PPIO as a dictionary for PPI annotation extraction. A PPIO-based approach which consists of three steps was proposed to accomplish the annotation task. First, a string matching algorithm[2] was applied to recognize all the case-insensitive names and synonyms of the PPIO terms in sentences containing PPIs. Then, in the case of multiple matches, the longest match was selected. For instance, when the terms “regulation” and “regulation of transcription” were both identified, “regulation of transcription” was selected. Finally, the results **were validated manually** and the performance of the PPIO-based approach was evaluated using the curated corpus described above. The evaluation process focused on the performance comparison between the automatically assigned corpus and the manually curated corpus.

## Evaluation metrices

Three commonly used metrics, precision, recall and F-score, are used to evaluate the performance of PPI extraction, as presented below by Equations 1, 2, and 3 respectively.

$$\text{Precision = }\frac{\text{True Positive}}{\text{True Positive}+\text{ False Positive}}\text{ ...... ...... ...... (1)}$$

$$\text{ Recall = }\frac{\text{ True Positive }}{\text{True Positive + False Negetive }}\text{ ... ...... ...... (2)}$$

$$\text{ F-score = }\frac{\text{ 2 }\times\text{ Precision }\times\text{ Recall }}{\text{Precision }+\text{ Recall }}\text{ ...... ......(3)}$$

where true positive is the number of entities that were found by the PPIO-based text mining system, and those matched the annotations in the curated corpus, false positive is the number of entities that were automatically assigned by the PPIO-based text mining system but could not be matched to any annotations in the manually curated corpus, and false negative is the number of entities that were not found by the PPIO-based approach when compared with the manually curated annotations. Higher precision, recall and F-score indicate high performance.

# Supplementary References

1. Krallinger M, Leitner F, Rodriguez-Penagos C, Valencia A: **Overview of the protein-protein interaction annotation extraction task of BioCreative II**. *Genome Biology* 2008, **9**(Suppl 2):S4.

2. Ivchenko O, Younesi E, Shahid M, Wolf A, Müller B, Hofmann-Apitius M: **PLIO: an ontology for formal description of protein–ligand interactions**. *Bioinformatics* 2011, **27**(12):1684-1690.
